# Chronic Inhibition of mROS Protects Against Coronary Endothelial Dysfunction in Mice With Diabetes

**DOI:** 10.3389/fcell.2021.643810

**Published:** 2021-02-18

**Authors:** Hang Xing, Zhiqi Zhang, Guangbin Shi, Yixin He, Yi Song, Yuhong Liu, Elizabeth O. Harrington, Frank W. Sellke, Jun Feng

**Affiliations:** ^1^Cardiothoracic Surgery Research Laboratory, Cardiovascular Research Center, Department of Surgery, Rhode Island Hospital, Alpert Medical School of Brown University, Providence, RI, United States; ^2^Vascular Research Laboratory, Providence VA Medical Center, Department of Medicine, Alpert Medical School of Brown University, Providence, RI, United States

**Keywords:** coronary endothelial function, mitochondrial reactive oxygen species, diabetes, potassium channels, coronary microcirculation

## Abstract

Diabetes is associated with coronary endothelial dysfunction. Persistent oxidative stress during diabetes contributes to coronary endothelial dysfunction. The mitochondria are main sources of reactive oxygen species (ROS) in diabetes, and mitochondria-targeted antioxidant mito-Tempo can prevent mitochondrial reactive oxygen species (mROS) generation in a variety of disorders. Inhibition/inactivation of small-conductance Ca^2+^-activated K^+^ (SK) channels contribute to diabetic downregulation of coronary endothelial function/relaxation. However, few investigated the role of mROS on endothelial dysfunction/vasodilation and endothelial SK channel downregulation in diabetes. The aim of present study was to investigate the chronic administration of mito-Tempo, on coronary vasodilation, and endothelial SK channel activity of mice with or without diabetes. Mito-Tempo (1 mg/kg/day) was applied to the mice with or without diabetes (*n* = 10/group) for 4 weeks. *In vitro* relaxation response of pre-contracted arteries was examined in the presence or absence of the vasodilatory agents. SK channel currents of the isolated mouse heart endothelial cells were measured using whole-cell patch clamp methods. At baseline, coronary endothelium-dependent relaxation responses to ADP and the selective SK channel activator NS309 and endothelial SK channel currents were decreased in diabetic mice compared with that in non-diabetic (ND) mice (*p* < 0.05). After a 4-week treatment with mito-Tempo, coronary endothelium-dependent relaxation response to ADP or NS309 and endothelial SK channel currents in the diabetic mice was significantly improved when compared with that in untreated diabetic mice (*p* < 0.05). Interestingly, coronary relaxation responses to ADP and NS309 and endothelial SK channel currents were not significantly changed in ND mice after mito-Tempo treatment, as compared to that of untreated control group. Chronic inhibition of endothelial mROS appears to improve coronary endothelial function/dilation and SK channel activity in diabetes, and mROS inhibitors may be a novel strategy to treat vascular complications in diabetes.

## Graphical Abstract

Chronic hyperglycemia during diabetes causes persistent overproduction of mitochondrial ROS (mROS), resulting in inhibition of endothelial SK channel activity, impairment of coronary endothelium-dependent relaxation and dysregulation of antioxidant/pro-cell death enzyme. Chronic inhibition of mROS with mito-Tempo prevents endothelial mROS production resulting in increasing endothelial SK currents, enhancing antioxidant enzyme production, and improving coronary endothelium-dependent relaxation.

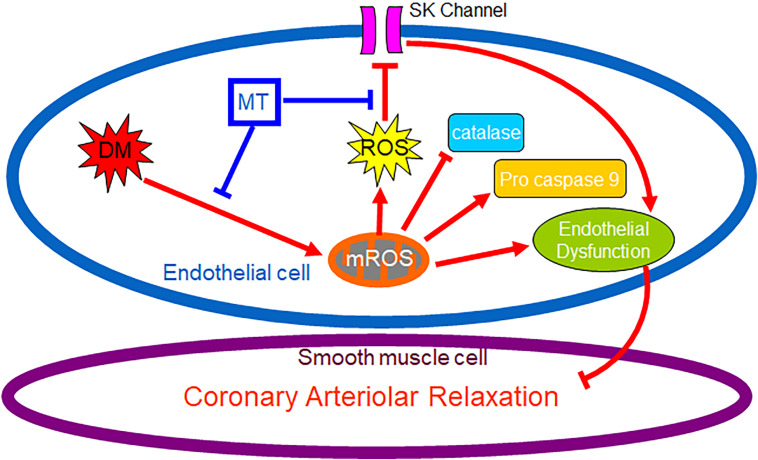

## Introduction

Macro- and micro-vascular diseases are the principal contributors to the increased morbidity and mortality associated with diabetes (DM) with the mortality rate of diabetic patients 3–4 times that of the general population (Haffner et al., 1998; Wilson et al., 2005). Diabetes is associated with coronary endothelial dysfunction (Emani et al., 2009; Ismail-Beigi et al., 2010; Feng et al., 2011, 2012a,b); a major risk factor of cardiovascular complications of DM. Given that DM affects 20% of the United States population and is increasing dramatically in prevalence, this remains a considerable clinical problem. Thus, investigation into mitochondrial reactive oxygen species (mROS) impact of diabetic regulation of endothelial function/protection in the coronary circulation with translational research model is a crucial step linking bench to bedside. Endothelial dysfunction from DM is associated with altered metabolism and inactivation of small conductance calcium-activated-potassium (SK) channels in the animal and human coronary vasculature (Makino et al., 2000; Ding et al., 2005; Burnham et al., 2006; Leo et al., 2011; Feng et al., 2012b, 2017; Liu et al., 2015, 2018, 2020). However, the precise mechanisms responsible for diabetic inactivation of SK and coronary endothelial dysfunction are still undefined.

Persistent oxidative stress during DM contributes to coronary endothelial dysfunction by reducing endothelial nitric oxide (eNOS) syntheses, increasing eNOS uncoupling (Si et al., 2006). The mitochondria are main sources of reactive oxygen species (ROS) in DM (Kizhakekuttu et al., 2012; Cho et al., 2013), and mitochondria-targeted antioxidants can prevent mROS generation in a variety of disorders (O’Malley et al., 2006; Graham et al., 2009; Galkin et al., 2014; Szczesny et al., 2014; Sada et al., 2016). However, few have evaluated the effects of chronic mROS inhibition on coronary endothelial function and SK channel activity *in vivo* in the setting of diabetes. Mito-Tempo, a mitochondria-targeted antioxidant, has been shown to reduce cellular levels of ROS, which shows promise in protecting cells against oxidative stress and cell damage (Park et al., 2016; Chen et al., 2017). However, its efficacy in preventing coronary endothelial and SK channel dysfunction in the setting of DM has not been investigated. Thus, we hypothesized that DM induces persistent overproduction of coronary endothelial mROS, resulting in impairment of coronary endothelium-dependent relaxation and dysfunction of endothelial SK channel. We further hypothesized that chronic inhibition of mROS during diabetes would suppress endothelial mROS overproduction resulting in improving coronary endothelium-dependent relaxation and enhancing endothelial SK channel function. Using genetically modified, obesity/type-2-DM mice, we investigated the effects of chronic treatment with mito-Tempo on coronary endothelium-dependent relaxation and endothelial SK channel function, as well as endothelial mROS production.

## Materials and Methods

### Animals

C57BL/6J mice (male, 12 weeks old) served as control of non-diabetic (ND) group (*n* = 20). Genetically modified mice exhibiting type-2 DM and obesity (BKS.Cg - *Dock7*^*m*^ +/+ *Lepr*^*db*^/J male, 12 weeks old, *n* = 20), were used in this study (Jackson Laboratory, Bar Harbor, Maine). All experiments were approved by the Institutional Animal Care and Use Committee of the Rhode Island Hospital.

### Mito-Tempo Treatment and Mouse Heart Harvesting

The mice were divided into following 4 groups (*n* = 10/group): (1) the untreated control ND mice received saline i.p. injection; (2) mito-Tempo treated ND mice received 1 mg/kg/day mito-Tempo i.p., injection; (3) the untreated DM mice saline i.p., injection; and (4) mito-Tempo treated DM mice received 1 mg/kg/day mito-Tempo i.p., injection. After 4 weeks of treatment, the mice were anesthetized using inhalant isoflurane and thoracotomy was performed and the heart was removed. The heart tissue was placed in cold Krebs buffer in preparation for *in vitro* microvascular study or preserved in cell culture medium for endothelial cell isolation or stored in liquid nitrogen for molecular analysis.

### Vascular Reactivity

After the mouse hearts (*n* = 6/group) were removed from the 4 experimental groups, they were immediately placed into cold (4°C) Krebs buffer [(in millimoles per liter): NaCl 119.0, NaHCO3 25.0, KCl 4.6, KH2PO4 1.2, MgSO_4_ 1.2, CaCl_2_ 1.8, and glucose 11.0]. The mouse small coronary (LAD) arteries (70–120 μm internal diameters) (Zhang et al., 2020) were dissected using a 10–60X dissecting microscope (Olympus Optical, Tokyo, Japan). Vessel studies were performed *in vitro* in a pressurized (40-mmHg) no-flow state using video-microscopy as previously described (Zhang et al., 2020). After 60-min period of equilibration, the vessel was pre-constricted with the thromboxane A2 analog U46619 (4 × 10^–7^–1 × 10^–6^ M) or endothelin-1 (10^–7^ M) and then receive the vasodilatory agents, sodium nitroprusside (SNP, 1 × 10^–9^–1 × 10^–4^ M), or adenosine 5′dephosphate (ADP, 1 × 10^–9^–1 × 10^–4^ M) or the selective SK channel activator NS309 (1 × 10^–9^–1 × 10^–4^ M), respectively (Feng et al., 2008; Liu et al., 2008, 2015, 2018; Zhang et al., 2020).

### Endothelial Cell Isolation and Culture

Mouse heart endothelial cells (MHECs) were isolated from the harvested heart of DM- and ND-mice (*n*=4/group), and cultured as previously described (Liu et al., 2015). MHECs (passage 0) were grown in the DMEM with 20% FCS + Pen/Strep + 100 μg/mL Heparin (Sigma) + 100 μg/mL ECGS (Biomedical Technologies, Stoughton, MA) + 1× non-essential amino acids + 2 mM L-glutamine + 1× sodium pyruvate + 25 mM HEPES in a humidified incubator with 5%CO_2_ at 37°C according to the manufacturer’s protocols and our previous study. Results were obtained, in triplicate, using three independent batches of isolation per group (Zhang et al., 2020).

### SK Channel Recording in MHECs

The cultured MHECs (passage 0) from mice with or without DM were washed twice with Ca^2+–^free DMEM, incubated with 0.05% trypsin and 0.02% EDTA for 1–2 min. An Axopatch-200B amplifier and pClamp 10.6 (Molecular Devices, Foster City, United States) were used to record and analyze K^+^ currents of MHECs in the whole-cell configuration in the voltage-clamp mode. The bath solution contained (in mM): 140 NaCl, 5 KCl, 1 CaCl_2_, 2 MgCl_2_, 10 HEPES, 30 glucose (pH 7.4). The patch pipette resistance was 1–3 MΩ and filled with the pipette solution contained (mM): 110 K-Aspartate, 20 KCl, 1 MgCl_2_, 8.5 CaCl_2_, 10 HEPES, 8 NaCl, 0.01 Niflumic acid and 10 BAPTA (pH 7.2, with calculated free Ca^2+^ 400 nM). The cells were examined every 5 s at the holding potential of −50 mV by 150 ms test pulses between −100 and +100 mV in 20 mV increments. Sampling rate was 10 kHz with low-pass filter set at 2 KHz. The effect of selective SK channel activator NS309 (10^–6^ M) on the whole cell K^+^ currents was examined. The specificity of NS309 was confirmed by simultaneous application of the selective SK (SK2/SK3) blocker apamin (10^–7^ M) and SK4 (IK) blocker TRAM34 (10^–5^ M) (Zhang et al., 2020).

### Measurement of mROS in the MHECs

Mouse heart endothelial cells were loaded with 5 μmol/L MitoSox Red and 100 nmol/L MitoTracker Green FM (Invitrogen) for 10 min at 37°C. Images were taken on a Zeiss LSM710 confocal microscope (Carl Zeiss GmbH, Germany) using an argon laser excitation (514 nm) with emission collection through a 560 nm long pass filter. The mean values of the whole cell fluorescence of MitoSOX^TM^ Red were obtained with ImageJ software (Liu et al., 2020).

### Immunoblot

The methods for whole cell protein purification, Western blotting and imaging quantification have been described previously. Membranes were incubated overnight at 4°C with primary antibodies against catalase and procaspase 9 (Cell Signaling, Danvers, MA, United States). After washing with TBST, membranes were incubated with the appropriate secondary antibody conjugated to horseradish peroxidase. All membranes were also incubated with GAPDH (glyceraldehyde-3-phosphate) or alpha-tubulin (Cell Signaling Tech. Danvers, MA, United States) as loading controls (Feng et al., 2017; Liu et al., 2020; Zhang et al., 2020).

### Statistical Analysis

Data are presented as the standard deviation (SD) or mean and standard error of the mean (SEM). Vascular responses are expressed as percent relaxation of the pre-constricted diameter. Data of vascular reactivity and channel current activity were analyzed using two-way repeated-measures ANOVA with a *post hoc* Bonferroni test. Other data were analyzed by either one-way ANOVA or Student’s *t*-test (GraphPad Software, Inc, San Diego, CA, United States). *P*-values < 0.05 were considered significant.

## Results

### Mice Characteristics

**FIGURE 1:**
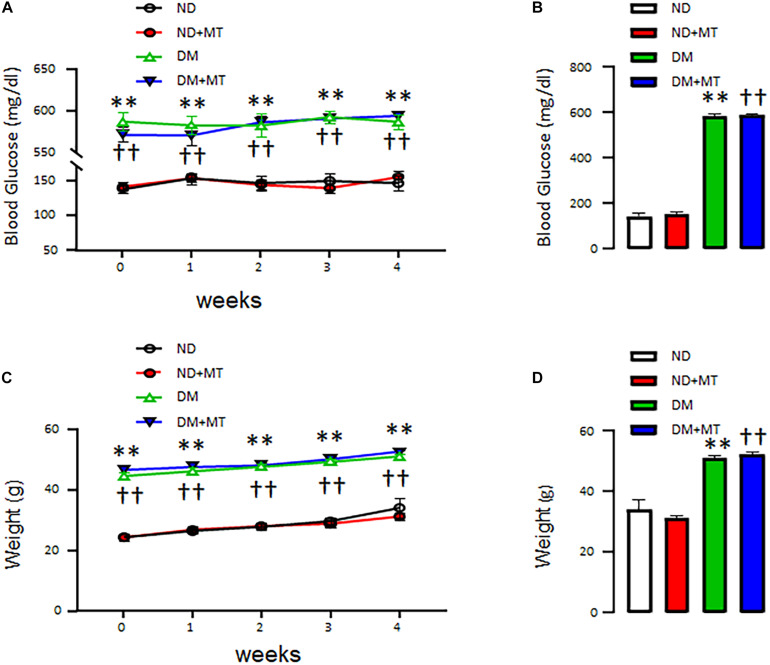
The effects of chronic treatment with mito-Tempo (MT) on body weight and blood glucose level in diabetic (DM) and non-diabetic (ND) mice. Mice (8 weeks of age) were intraperitoneal injection mito-Tempo (1 mg/kg) for 4 weeks; DM + MT = diabetic mice treated with mito-Tempo for 4 weeks, ND + MT = non-diabetic mice treated with mito-Tempo for 4 weeks; **(A)** Blood glucose level of DM mice was significantly higher than ND mice (*p* < 0.01), chronic MT treatment had no effect on body weight in both mice (*p* > 0.05), **(B)** Bar graph showing blood glucose of DM and ND mice after 4 week treatment with MT, **(C)** Body weight of DM mice was significantly higher than ND mice (*p* < 0.01), chronic treatment with MT had no effect on body weight in both mice (*p* > 0.05), and **(D)** Bar graph showing body weight of DM and ND mice after 4 weeks chronic treatment of MT; ^∗∗^*p* < 0.01 vs ND mice; ^††^*p* < 0.01 vs. ND + MT.

The effects of DM and 4-week treatment with mito-Tempo on mouse body weight and blood glucose levels are summarized in Figure 1. At baseline, the body weight (Figure 1A) and blood glucose (Figure 1B) of genetically modified DM mice were higher than that of ND mice (*p* < 0.0001). Four-week treatment with mito-Tempo did not change the body weight and blood glucose levels, as compared to their non-treated controls (*P* > 0.05), respectively.

### The Effects of DM and Mito-Tempo Treatment on Small Coronary Arterial Relaxation

**FIGURE 2:**
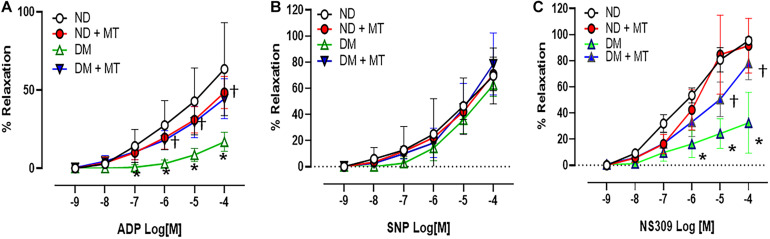
Effects of chronic treatment with mito-Tempo (MT) on mouse small coronary arterial relaxation in responses to vasodilators. **(A)** Mouse small coronary arterial relaxation responses to endothelium-dependent vasodilators ADP in diabetic (DM) and non-diabetic (DM) mice after 4-week treatment with mito-Tempo (1 mg/kg/day), DM + MT = diabetic mice treated with mito-Tempo for 4 weeks, ND + MT = non-diabetic mice treated with mito-Tempo for 4 weeks; **(B)** Mouse small coronary arterial relaxation responses to endothelium-independent vasodilators SNP in ND and DM mice after chronic treatment with mito-Tempo, *n* = 5–6/group. **(C)** Mouse small coronary arterial relaxation responses to the selective SK channel activator NS309 in ND and DM mice and after chronic treatment with MT, *n* = 6–8/group, *p < 0.05 ND vs.DM, †*p* < 0.05 DM + mito-Tempo vs. DM.

The endothelium-independent vasodilator SNP, the endothelium-dependent vasodilator ADP, and the endothelium-dependent/SK channel activator NS309 induced dose-dependent relaxation response in the mouse small coronary arteries (Figure 2). There were no significant differences in the responses to the endothelium-independent vasodilator SNP between ND and DM mice or mito-Tempo treated groups vs. control untreated groups (Figure 2). However, in the control untreated (saline) groups, DM significantly reduced the relaxation response to ADP and NS309 compared to that of ND group (*p* < 0.05). Four-week treatment with mito-Tempo significantly improved relaxation response to ADP and NS309 in the DM vessels, as compared with that in untreated DM vessels (*p* < 0.05). Interestingly, 4-week treatment with mito-Tempo failed to affect the relaxation response to ADP and NS309 of small coronary arteries in ND mice, as compared to the untreated ND mice (*p* > 0.05).

### The Effects of DM and Mito-Tempo Treatment on Endothelial SK-Currents of MHECs

**FIGURE 3:**
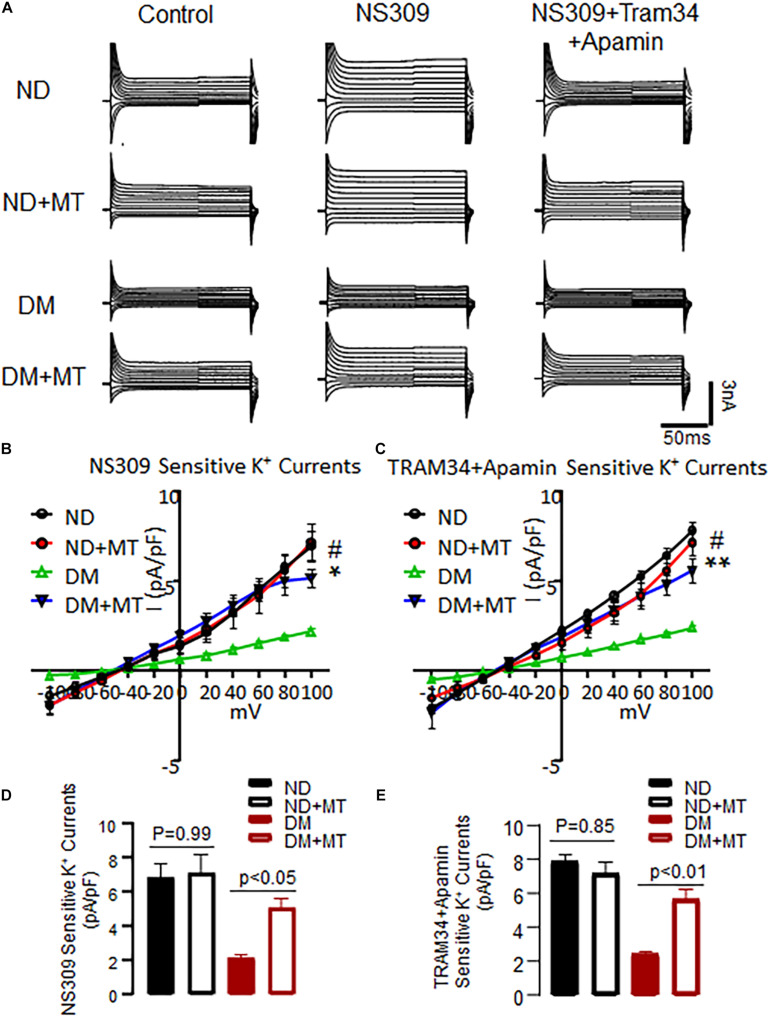
Chronic treatment with mito-Tempo (MT) significantly increased SK channels current in mouse heart endothelial cells (MHECs) of diabetics (DM) and non-diabetics (ND). **(A)** Representative traces of the whole cell currents of MHECs treated with or without MT. **(B)** Four-week treatment with MT increased SK channels current in responses to NS309 in MHECs of DM, *n* = 6/group, *p < 0.05 DM + MT vs. DM, #*p* < 0.01 ND vs. DM, DM + MT = diabetic mice treated with mito-Tempo for 4 weeks, ND + MT = non-diabetic mice treated with mito-Tempo for 4 weeks; **(C)** NS309-sensitive K^+^ currents were blocked by simultaneous co-application of the selective SK2/SK3 blocker Apamin and SK4 blocker TRAM34. *n* = 6/group, **p < 0.01 DM + MT vs. DM, #*p* < 0.01 ND vs. DM. **(D,E)** Cumulative bar graph of NS309-activated currents and TRAM34 + Apamin blocked currents at +100 mV showing that was significantly increased in DM + MT MHECs compared with DM MHECs. *n* = 5/group.

The basal endothelial K^+^ currents were significantly diminished in the MHECs with DM compared to ND (Figure 3, *p* < 0.05). Application of the SK channel activator NS 309 enhanced endothelial SK current in both of ND and DM cells, however, the response to NS309 was significantly diminished in the DM mice compared to that of ND (Figure 3). Four-week treatment with mito-Tempo significantly enhanced endothelial K^+^ currents in the DM cells compared to untreated DM cells (Figure 3, *p* < 0.05). Subsequent application of SK blockers, apamin (10^–7^ M) and TRAM34 (10^–6^ M), prevented NS309-indued effects on K^+^ currents in both ND and DM groups (Figure 3). In contrast, 4-week treatment with mito-Tempo failed to affect endothelial K^+^ currents in the ND mice (*p* > 0.05).

### The Effects of DM and Mito-Tempo Treatment on mROS in MHECs

**FIGURE 4:**
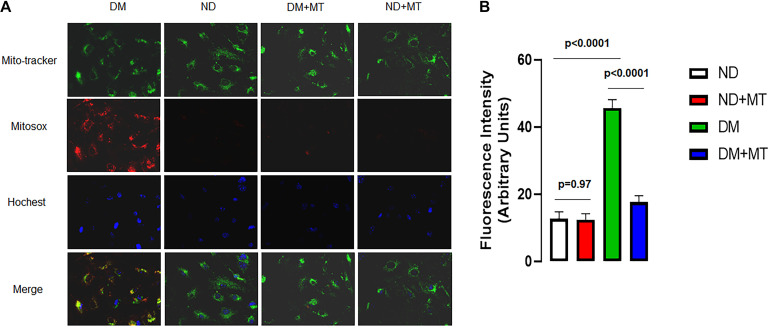
The effects of 4-week treatment with mito-Tempo (1 mg/kg/day) on the production of mitochondrial ROS (mROS) in the mouse heart endothelial cells (MHECs) from diabetic (DM) and non-diabetic (ND) mice, **(A)** MHECs were loaded with mitochondrial marker mitotracker (green) and mROS marker (red), and Hochest 33342 (blue); **(B)** bar graph showing the analysis of fluorescence intensity (mROS signals) in the experimental groups. *n* = 4/group. DM + mito-Tempo = diabetic mice treated with mito-Tempo for 4 weeks, ND + mito-Tempo = non-diabetic mice treated with mito-Tempo for 4 weeks.

The untreated MHECs of DM mice had significantly higher levels of mROS than their ND counterparts (Figure 4, *p* < 0.05). In the DM groups, 4-week treatment with mito-TEMPO significantly reduced mROS (*p* < 0.05) compared to untreated DM. In contrast, in the ND groups, 4-week treatment with mito-TEMPO did not affect mROS levels compared to untreated ND (*p* < 0.05).

### The Effects of DM and Mito-Tempo Treatment on Catalase and Pro-caspase 9 Expression

**FIGURE 5:**
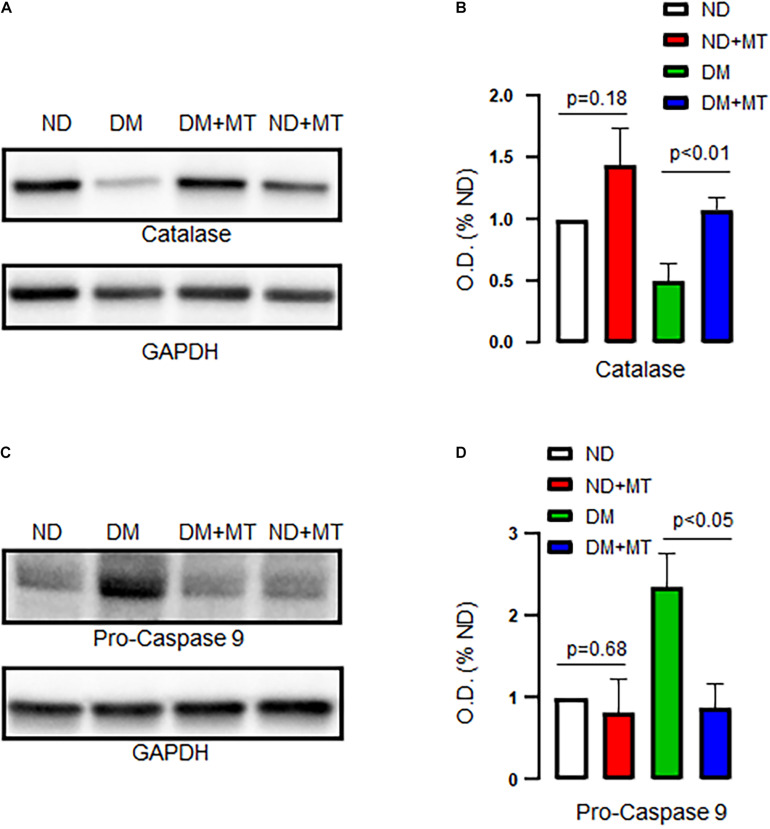
Effects of chronic treatment with mito-Tempo (MT) on catalase and pro-caspase 9 in ND and DM heart tissue samples. **(A)** Representative immunoblots for catalase. **(B)** Densitometric analysis of signal intensity in the catalase protein expression of heart tissue samples in DM and ND mice treated with or without mito-Tempo; DM + MT = diabetic mice treated with mito-Tempo for 4 weeks, ND + MT = non-diabetic mice treated with mito-Tempo for 4 weeks; *n* = 6/group; **(C)** Representative immunoblots of ECs for pro-caspase 9. **(D)** Densitometric analysis of signal intensity in the pro-caspase 9 protein expression of heart tissue samples in DM and ND mice after chronic treatment with mito-Tempo. *n* = 6/group.

DM significantly decreased protein expression of catalase but enhanced pro-caspase 9 in the LV myocardium compared to that of ND (Figure 5, *p* < 0.05), respectively. Treatment of the diabetic mice with mito-Tempo significantly increased protein expression of catalase but decreased pro-caspase 9 in the DM heart compared to that of untreated diabetic mice (*p* < 0.05), respectively. In contrast, chronic treatment with mito-Tempo failed to affect catalase and pro-caspase 9 expression in the ND mice.

## Discussion

We and others have previously reported that DM is associated with coronary endothelial dysfunction in animals and humans (Feng et al., 2012a; Liu et al., 2015, 2018, 2020; Zhang et al., 2020). The current study confirmed these previous findings by demonstrating that DM reduced mouse coronary endothelium-dependent relaxation/function. ROS generated during diabetic hyperglycemia are implicated in the development of diabetic vascular complications (Baynes, 1991; Li and Shah, 2004). Elevated levels of oxygen-derived free radicals are the initial source of endothelial dysfunction in DM. ROS not only reduce nitric oxide (NO) bioavailability via uncoupled eNOS, but also facilitate the production and/or action of endothelium-dependent constricting factors (EDCFs) (Srinivasan et al., 2013). Thus, the endothelial balance tips toward vasoconstrictor responses over the course of diabetes (Shi and Vanhoutte, 2009). Mitochondrial dysfunction plays a key role in endothelial dysfunction in DM. Emerging evidence shows that mitochondria are the dominant mechanisms of ROS production in the diabetic heart/vessels (Shenouda et al., 2011; Kizhakekuttu et al., 2012; Liu et al., 2020). The current study further indicates that DM significantly increases endothelial mROS in the MHECs. Four-week application of mito-Tempo in the mice with DM prevented mROS overproduction in MHECs and improved coronary endothelial function. Consistent with previous studies in animals and humans (Ding et al., 2005; Burnham et al., 2006; Leo et al., 2011; Liu et al., 2015, 2020; Zhang et al., 2020), in the current study we confirmed that DM decreased coronary relaxation response to the SK activators and endothelial SK current activity, suggesting that diabetic inhibition of SK channels may contribute to coronary endothelial dysfunction.

There are several novel findings in the current study. First, chronic treatment with mito-Tempo in mice *in vivo* caused significant reduction in coronary endothelial mROS, and improved coronary endothelial function as demonstrated by enhanced coronary relaxation response to endothelium-dependent vasodilator ADP. Second, chronic treatment with mito-Tempo *in vivo* significantly increased the relaxation response of diabetic coronary vasculature by the selective SK activator NS309. Third, chronic treatment with mito-Tempo improved SK current activity of MHECs in the diabetic mice. Finally, chronically treating the healthy mice with mito-Tempo did not affect coronary endothelial function and SK channel activity, suggesting that mROS play little role during normal condition. This finding was confirmed by the results that there were few endothelial mROS tested in the normal MHECs in mice without diabetes. Under physiological condition, endothelial cells largely depend on cytosolic glycolysis for generation of ATP and for its energy supply, which does occur in the mitochondria.

Endothelial cells release endothelium-derived relaxing factors (EDRF), such as NO, prostacyclin (PGI_2_), endothelium-dependent hyperpolarizing factors (EDHF) (Vanhoutte and Shimokawa, 1989; Feletou and Vanhoutte, 2009). SK channels are largely responsible for coronary arteriolar relaxation mediated by EDHF (Vanhoutte and Shimokawa, 1989; Feletou and Vanhoutte, 2009). Previous studies have demonstrated that ADP induced coronary vasodilation is endothelium- and NO-dependent (Vanhoutte and Shimokawa, 1989; Myers et al., 1992; Faraci et al., 2004). Inhibition of mROS with mito-Tempo in the diabetic mice improved coronary relaxation in response ADP, suggesting that mito-Tempo may potentiate endothelial eNOS coupling. In addition, we and others have observed that the selective SK activator NS309 induced coronary dilation partially via NO signaling pathway (Sheng et al., 2009; Dalsgaard et al., 2010; Zhang et al., 2020), suggesting that there is potential cross-talk between NO and EDHF in the endothelium. It can be assumed that mito-Tempo treatment may augment the cross talk between NO and EDHF in the endothelium leading to NS309-enhancemenet of coronary dilatation and SK channel activation in the diabetic mice.

Compelling evidences suggest that diabetes and hyperglycemia inhibits large-conductance Ca^2+^-activated K^+^ channel (BK) and voltage-gated K^+^ channel (Kv) function on vascular smooth muscle cells (Liu et al., 2001; Lu et al., 2006). This effect is mediated by direct oxidation of key cysteine residues in the bowl region of this channel (Tang et al., 2004; Lu et al., 2006), inhibition of Akt signaling, facilitation of the FOXO-3a/FBXO-dependent BK-β1 degradation (Lu et al., 2006, 2012), and other oxidation of vascular K^+^ channels, such as peroxynitrite (Liu et al., 2002; Cheng et al., 2011). However, mechanisms responsible for diabetic downregulation of endothelial SK channels are still largely unknown. A recent study also indicates that diabetic oxidation and nitration of endothelial SK channels may be one of the potential mechanisms (Cheng et al., 2018). Interesting, we recently observed that diabetic dysregulation of pyridine nucleotide NADH may also contribute to the inactivation of coronary endothelial SK channels (Liu et al., 2020). Furthermore, the enhancement of antioxidant enzyme expression in the diabetic heart may account for endothelial protective effects of chronic mROS inhibition.

However, some studies suggested that ROS or H_2_O_2_ may be some of EDHFs contributing to the maintenance of vascular relaxation in settings of metabolic syndrome. Acute administration of H_2_O_2_ (high dose) can cause vasodilation, whereas catalase can block H_2_O_2_ induced vasodilation (Matoba et al., 2002; Hatoum et al., 2005). This discrepancy between the previous studies and the current findings may be due to the animal models used in the studies; that is acute metabolic syndrome versus chronic diabetes. Thus, the data suggest that persistent overproduction of ROS is detrimental for coronary endothelial function during chronic hyperglycemia or chronic diabetes.

There are still some limitations in the current study. For example, we did not measure SK protein expression in the mouse heart after mito-Tempo treatment. It has been established that SK2, SK3, and SK4 are predominantly expressed in the mouse heart (Tuteja et al., 2005). SK2 and SK3 mainly present in the cardiomyocytes, whereas SK3 and SK4 (IK) in the endothelial cells. We have recently observed that there were no significant differences in protein expression of SK3 and SK4 between ND and DM MHECs (Zhang et al., 2020) or between patients with ND and patients with DM in the atrial myocardium/coronary endothelial cells, (Liu et al., 2015, 2018, 2020) suggesting that DM may affect endothelial SK gating or trafficking instead of the total protein levels. However, we did not conclusively distinguish the effects of diabetes and mito-Tempo on SK expression in the mouse heart endothelial and cardiomyocytes in the current study.

This is the first study to observe that the activation of endothelial SK channels may contribute to mROS inhibition-induced protection of coronary endothelial function/relaxation. Our study strongly suggests that persistent over-production mROS causes coronary endothelial dysfunction and chronic inhibition of mROS may provide therapeutic effects on diabetic complications.

## Data Availability Statement

The raw data supporting the conclusions of this article will be made available by the authors, without undue reservation.

## Ethics Statement

The animal study was reviewed and approved by the Institutional Animal Care and Use Committee of the Rhode Island Hospital.

## Author Contributions

HX carried out ion channel recoding experiments, all the data analysis, and draft the primary manuscript. ZZ, YS, and YL performed animal handling, *in vitro* vessel reactivity experiments and related data analysis. GS, YH, and EH performed cell culture, mROS, and protein analysis. JF, EH, and FS obtained funding, critical revision and supervised the project. All authors were involved in writing and editing the manuscript.

## Conflict of Interest

The authors declare that the research was conducted in the absence of any commercial or financial relationships that could be construed as a potential conflict of interest.
