# Supplementary material for: Chronic Inhibition of mROS Protects Against Coronary Endothelial Dysfunction in Mice With Diabetes
Source: Front Cell Dev Biol. 2021 Feb 18;9:643810. doi: 10.3389/fcell.2021.643810 (PMC7930489; doi:10.3389/fcell.2021.643810)
Supplement: Supplementary file 1 [file Data_Sheet_1.PDF]

Supplemental Files

Isolation of Mouse Heart Endothelial Cells (MHECs)

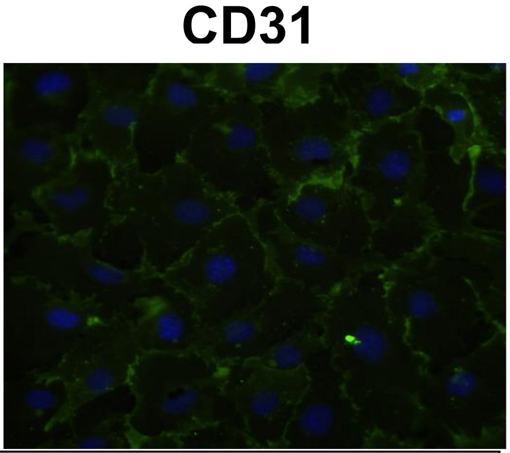

Fig.1S. Endothelial cells (green) were confirmed by immuno-histochemical staining of the endothelial marker CD31, Blue, nuclei

MHECs were isolated from the harvested heart of mice with or without type-2 DM, as previously described.[1-3] For each experiment, primary cultures were started simultaneously (a pool of 5 cases per group). The isolated heart was placed in a 15cm dish containing 15 mL of cold DMEM medium, cut into 2-3 pieces and transferred heart pieces in 25 mL of pre-warmed, 37°C Collagenase (2 mg/mL), incubate at 37°C with gentle agitation for 45 min. The cell suspension was triturated and Pipetted through a 70 um disposable cell strainer (Falcon #35 2350) into a 50mL conical tube and then wash sieve with 10 mL of Medium. The cell suspension was then spined down at 400g (1300 rpm in GH3.7 rotor) for 8 min at 4°C and then re-suspended in 2 mL of cold PBS + 0.1% BSA. The nucleated cells were counted on a Coulter counter by adding 5uL of suspension to 10mL of Isotonic buffer plus 3 drops of Zap-globin. MHECs (passage 0) were grown in the DMEM with 20%FCS + Pen/Strep + 100µg/mL Heparin (Sigma) + 100 µg/mL ECGS (Biomedical Technologies, Stoughton, MA) +1x nonessential amino acids + 2mM L-glutamine + 1x sodium pyruvate + 25mM HEPES in a humidified incubator with 5%CO_2 at 37°C according to the manufacturer's protocols and our previous study.[1-3] CD31 positive cells (green) have been shown in Fig. 1S to confirm the isolated endothelial cells..

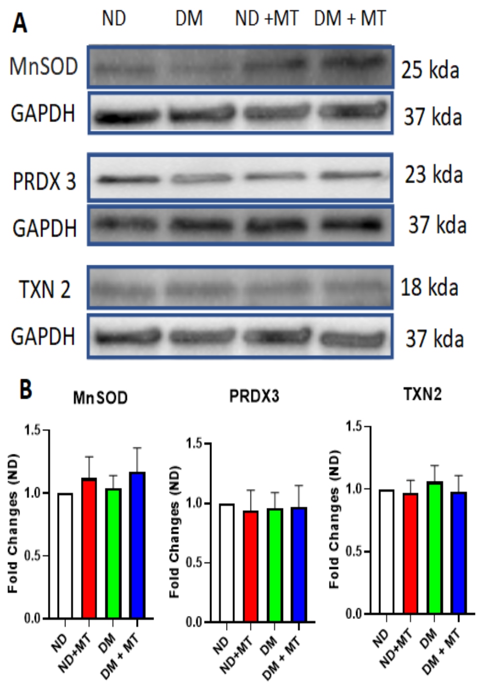

Fig.2S. Effects of chronic treatment with mito-Tempo (MT) on protein expression of MnSOD, PRDX3 and TXN2 in ND and DM heart tissue samples. A. Representative immunoblots for of MnSOD, PRDX3 and TXN2. B. Densitometric analysis of signal intensity in of MnSOD, PRDX3 and TXN2 protein expression of heart tissue samples in DM and ND mice treated with or without mito-Tempo; DM + MT = diabetic mice treated with mito-Tempo for 4 weeks, ND + MT = non-diabetic mice treated with mito-Tempo for 4 weeks, n = 3/group. ND vs. DM : no significance, ND vs. ND+MT: no significance, DM vs. DM+MT : no significance

MnSOD, PRDX3 and TXN2 Protein Expression

The methods for measuring the protein expression of MnSOD (manganese-dependent superoxide dismutase), PRDX3 (peroxiredoxin-3, thioredoxin-dependent peroxide reductase) and TXN2 (Thioredoxin-2, a redox-active protein) has been described in the manuscript. Mito-Tempo treatment tends to increase MnSOD expression in both ND and DM mice, but failed to reach statistical significance (Fig.2S). There were no significant

differences in PRDX3 and TXN2 protein expression between ND and DM, or Mito-Tempo treated and untreated groups (P>0.05), respectively.

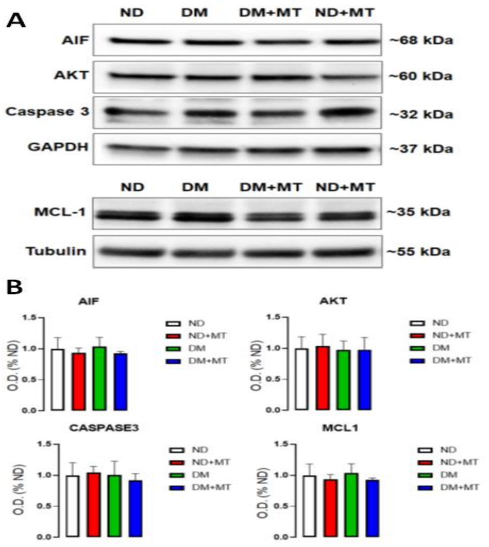

Fig.3S. Effects of chronic treatment with mito-Tempo (MT) on protein expression of AIF, AKT, caspase 3 and MCL-1 in ND and DM heart tissue samples. A. Representative immunoblots for AIF, AKT, caspase 3 and MCL-1. B. Densitometric analysis of signal intensity in AIF, AKT, caspase 3 and MCL-1 protein expression of heart tissue samples in DM and ND mice treated with or without mito-Tempo; DM + MT = diabetic mice treated with mito-Tempo for 4 weeks, ND + MT = non-diabetic mice treated with mito-Tempo for 4 weeks, n = 3-5/group. ND vs. DM : no significance, ND vs. ND+MT: no significance, DM vs. DM+MT : no significance

AIF, AKT, Caspase 3 and MCL-1 Protein Expression

The methods of Western Blotting for measuring the pro-apoptosis protein expression has been described in the manuscript. There were no significant differences in the protein expression of AIF, AKT, caspase 3 and MCL-in the mouse heart between ND and DM, or mito-Tempo treated and untreated groups, respectively (Fig.3S).

References:

1. Shafique E, Torina A, Reichert K, Colantuono B, Nur N, Zeeshan K, Ravichandran V, Liu Y, Feng J, Benjamin LE, Irani K, Harrington EO, Sellke FW, Abid MR. Mitochondrial redox plays a critical role in the paradoxical effects of napdh oxidase-derived ros on coronary endothelium. *Cardiovascular research*. 2017;113:234-246
2. Shafique E, Choy WC, Liu Y, Feng J, Cordeiro B, Lyra A, Arafah M, Yassin-Kassab A, Zanetti AV, Clements RT, Bianchi C, Benjamin LE, Sellke FW, Abid MR. Oxidative stress improves coronary endothelial function through activation of the pro-survival kinase ampk. *Aging*. 2013;5:515-530
3. Zhang ZQ, Shi GB, Liu YH, Xing H, Kabakov A, Zhao AS, Agbortoko V, Kim J, Koren G, Singh AK, Harrington EO, Sellke FW, Feng J. Coronary endothelial dysfunction prevented by small conductance calcium activated potassium channel activator in mice and patients with diabetes. Journal of Thoracic and Cardiovascular Surgery. 2020 Feb 19:S0022-5223(20)30426-8. doi: 10.1016/j.jtcvs.2020.01.078. Online ahead of print. PMCID: PMC7439127
